# Association of *PTGER4* and *PRKAA1* genetic polymorphisms with gastric cancer

**DOI:** 10.1186/s12920-023-01645-1

**Published:** 2023-09-05

**Authors:** Shuyong Yu, Ruisha Tu, Zhaowei Chen, Jian Song, Ping Li, Feixiang Hu, Guihong Yuan, Ronglin Zhang, Yini Li

**Affiliations:** 1 Department of Gastrointestinal Surgery, Hainan Cancer Hospital, Haikou, Hainan, 570312 China; 2 Department of Gastroenterology, Southern University of Science and Technology Hospital, Shenzhen, 518055 China; 3 Department of Digestive Endoscopy, Hainan Cancer Hospital, Haikou, Hainan, 570312 China

**Keywords:** Gastric cancer, *PTGER4* polymorphisms, *PRKAA1* polymorphisms, Stratification analysis

## Abstract

**Background:**

Gastric cancer (GC) is one of the most common malignancies, affected by several genetic loci in the clinical phenotype. This study aimed to determine the association between *PTGER4* and *PRKAA1* gene polymorphisms and the risk of GC.

**Methods:**

A total of 509 GC patients and 507 age and sex-matched healthy controls were recruited to explore the association between *PTGER4* and *PRKAA1* genetic polymorphisms and GC susceptibility. Logistic regression analysis was used to study the correlation between these SNPs and GC, with odd ratio (OR) and 95% confidence interval (CI) as indicators. Multifactor dimensionality reduction was utilized to analyze the genetic relationships among SNPs. was conducted to predict gene expression, the impact of SNPs on gene expression, and the signaling pathways involved in *PTGER4* and *PRKAA1*.

**Results:**

Overall, rs10036575 in *PTGER4* (OR = 0.82, *p* = 0.029), rs10074991 (OR = 0.82, *p* = 0.024) and rs13361707 (OR = 0.82, *p* = 0.030) in *PRKAA1* were associated with susceptibility to GC. Stratification analysis revealed that the effects of these SNPs in *PTGER4* and *PRKAA1* on GC susceptibility were dependent on smoking and were associated with a reduced risk of adenocarcinoma (*p* < 0.05). Bioinformatics analysis showed an association between SNPs and corresponding gene expression (*p* < 0.05), and *PRKAA1* may affect GC by mediating RhoA.

**Conclusion:**

This study suggests that *PTGER4* and *PRKAA1* SNPs might affect the susceptibility of GC, providing a new biological perspective for GC risk assessment, pathogenesis exploration, and personalized treatment.

## Introduction

Gastric cancer (GC) is a common malignancies worldwide, with over one million new cases each year and ranking as the fourth leading cause of cancer-related death [1, 2]. In the Nordic countries, GC has an estimated heritability of 22%, with multiple genetic pathogenic mutations in high penetrance genes [3]. It is estimated that there will be 10 million new GC cases and 5.6 million GC deaths in China between 2021 and 2035 [4]. GC is a highly aggressive malignancy with heterogeneity, posing a significant global health burden [5]. Its specific pathogenesis remains unclear, with both environmental and genetic factors playing a role in its occurrence and development [6]. Recent studies have identified several risk factors associated with GC, including age, gender, obesity, cigarette smoking, alcohol consumption, diet, and Helicobacter pylori infection [7–9]. Furthermore, genetic factors are believed to play a significant role in GC pathogenesis [10]. Single nucleotide polymorphisms (SNPs) are the most common form of gene mutation in the human genome. Genome-wide association studies have identified several GC susceptibility loci [11, 12].

*PRKAA1* is a gene e that encodes adenosine monophosphate-activated protein kinase (AMPK), a central metabolic switch involved in various diseases related to energy metabolism, particularly cancer [10, 13]. AMPK has been found to play a role in tumorigenesis and development through regulatory pathways [14]. Interestingly, *PRKAA1* promotes tumorigenesis and invasion, and its gene polymorphisms may be involved in the occurrence and development of GC by affecting energy metabolism regulation [14, 15]. Among these polymorphisms, the association between *PRKAA1* polymorphisms (rs13361707 and rs10074991) and GC risk has been extensively studied [16–18]. However, there is limited validation of the relationship between *PRKAA1* polymorphisms and GC risk in the Chinese Han population.

*PTGER4* is a G-protein-coupled receptor that mediates the action of prostaglandin E2 (PGE2), playing a crucial role in cancer cell proliferation, invasion, stem cell regeneration, and tumor angiogenesis [19]. *PTGER4* also plays a significant role in regulating cell migration and immune response [20, 21]. Studies have shown that *PTGER4* is involved in the growth and development of carcinomas, including colorectal cancer [19, 22–24]. Studies have shown that *PTGER4* is involved in the growth and development of carcinomas, including colorectal cancer [25]. However, there have been few studies on *PTGER4* expression in GC, and the detailed biological mechanism of its role in tumor cells remains poorly understood. The role of *PTGER4* polymorphism in GC function is also unknown. Therefore, further research on the role of *PTGER4* gene polymorphism in GC is essential. A comprehensive study of the relationship between *PTGER4* gene polymorphism and GC susceptibility can provide a foundation for the diagnosis and treatment of GC.

This study aims to investigate the relationship between *PTGER4* and *PRKAA1* SNPs and the risk of GC, examining the roles of *PTGER4* and *PRKAA1* genes in the occurrence and development of GC. A deeper understanding of GC pathogenesis is crucial for early detection, identification of risk factors, and personalized treatment.

## Methods

### Study subjects

A total of 1,016 subjects (509 GC cases and 507 healthy controls) were enrolled in this study to investigate the association between the *PTGER4* and *PRKAA1* genes polymorphisms and the risk of GC. The diagnosis of GC patients was confirmed through histopathological analysis and pathological data. Patients with a prior history of cancer, chemotherapy or radiotherapy, and immune system disorders were excluded from the case group. Blood samples were collected from patients prior to each treatment period. The control group was randomly selected from healthy volunteers who had no history of cancer. All participants provided written informed consent. Demographic data (age, gender, body mass index (BMI), smoking and drinking status) and clinical data (lymph node metastasis, staging, and adenocarcinoma status) were obtained through questionnaire surveys and hospital records. Definition of smoking and drinking status: Non-smokers/non-drinkers: Non-smokers/non-drinkers are participants who have never smoked or drank, or have only occasionally smoked or drank without developing a sustained habit. Smokers/drinkers are participants who continue to engage in smoking or drinking behavior and have developed a sustained habit of smoking or drinking.

### SNP selection and genotyping

The physical location of the *PTGER4* and *PRKAA1* genes was searched using NCBI database (https://www.ncbi.nlm.nih.gov/gene/). SNPs within 500 kb of *PTGER4* and *PRKAA1* genes with minor allele frequency (MAF) > 0.05, Hardy-Weinberg equilibrium (HWE) > 0.05, min genotype frequency > 75%, and r^2^ > 0.8 in the Chinese Han Beijing (CHB) population of 1000 Genome Project were screened using the VCF to PED Converter window (http://grch37.ensembl.org/HomoSapiens/Tools/VcftoPed) and Haploview software. Finally, Based on primer design and genotyping results, a total of nine SNPs (rs4613763, rs6880778, rs11742570, rs9292777, rs7725052, rs12186979, and rs10036575 in *PTGER4*, rs10074991 and rs13361707 in *PRKAA1*) were chosen for the association analysis. The functional annotation of SNPs was predicted using the Regulomedb database (https://regulomedb.org/). Peripheral blood genomic DNA was extracted using GoldMag DNA Purification Kit (GoldMag Co. Ltd.). The concentration and purity of DNA are detected using NanoDrop 2000 (Thermo Scientific). The Agena MassARRAY platform (Agena Bioscience, San Diego, CA, USA) was used for SNPs genotyping. AgenaTyper 4.0 software was used to organize and analyze genotype data.

### Bioinformatics analysis

Bioinformatics analysis was conducted using various databases. The Ualcan database (https://ualcan.path.uab.edu/analysis.html) was used to compare the expression of *PTGER4* and *PRKAA1* genes in stomach adenocarcinoma (STAD) and normal tissues. The GTEx Portal database (https://gtexportal.org/home/) predicted the association between SNPs and the expression levels of *PTGER4* and *PRKAA1* in gastric tissue. The GEO database (https://www.ncbi.nlm.nih.gov/gds) was utilized to analyze the relationship between GC and the expression levels of *PTGER4* and *PRKAA1* genes using the GSE26309 dataset. The STRING database (https://www.string-db.org/) was used to identify the interaction between PTGER4 and PRKAA1-related proteins. KEGG (Kyoto Encyclopedia of Genes and Genomes) [26] pathway enrichment analysis (https://www.kegg.jp/kegg/kegg1.html) and key target regulatory pathways were performed using the oebiotech platform (https://cloud.oebiotech.com/task/).

### Statistical analysis

Statistical analysis was conducted using SPSS (version 25), PLINK (version 1.9), and multifactor dimensionality reduction (MDR, version 3.0.2) software. A *p*-value < 0.05 was considered statistically significant. Logistic regression analysis was used to assess the correlation between genetic variations and the risk of GC, with odds ratios (OR) and 95% confidence intervals (CI) as indicators. Baseline data of controls and GC case groups were matched using student’s t-test and χ^2^ test. The genotype distributions in controls were assessed for Hardy-Weinberg equilibrium using the χ^2^ test.

## Results

### Subjects characteristics

In this study, a total of 1,016 subjects (509 GC cases and 507 controls) of the Han ethnicity from Hainan province were enrolled using a case-control experimental design. Table 1 provides a summary of the demographic characteristics and clinical information of the participants. The GC case group (61.35 ± 8.84) consisted of 382 males (75%) and 127 females (25%), and the control group (61.12 ± 11.33) consisted of 379 males (75%) and 128 females (25%). Among the participants, 279 cases (55%) were over the age of 60 years, and 325 cases (62%) were in the control group. There were no significant differences in terms of age (*p* = 0.712), gender (*p* = 0.913), smoking (*p* = 0.333), and drinking (*p* = 0.063) distributions between the control and GC case groups. Furthermore, it was observed that 314 (62%) patients had adenocarcinoma, 235 (46%) patients had lymph node metastasis, and 239 (47%) patients were in stage III-IV.

**Table 1:** Characteristics of patients with GC and health controls

Variable		Cases (n = 509)	Controls (n = 507)	*p*
Age	Mean ± SD, years	61.35 ± 8.84	61.12 ± 11.33	0.712
	> 60 years	279 (55%)	315 (62%)	
	≤ 60 years	230 (45%)	192 (38%)	
Gender	Male	382 (75%)	379 (75%)	0.913
	Female	127 (25%)	128 (25%)	
Smoking	Yes	233 (56%)	114 (22%)	0.333
	No	270 (53%)	172 (34%)	
	Unavailable	6 (1%)	221 (44%)	
Drinking	Yes	133 (26%)	119 (23%)	0.063
	No	357 (70%)	142 (28%)	
	Unavailable	19 (4%)	246 (49%)	
BMI	> 24 kg/m^2^	72 (14%)	183 (36%)	< 0.001
	≤ 24 kg/m^2^	401 (79%)	170 (34%)	
	Unavailable	36 (7%)	154 (30%)	
Lymph nodes metastasis	Yes	235 (46%)		
	No	97 (19%)		
	Unavailable	177 (35%)		
Stage	I-II	109 (21%)		
	III-IV	239 (47%)		
	Unavailable	161 (32%)		
Adenocarcinoma	Yes	314 (62%)		
	No	195 (38%)		

SD: standard deviation; BMI: body mass index

*p* values were calculated by χ^2^ test or the Student’s t test

*p* < 0.05 indicates statistical significance

### Genetic characteristics of selected SNPs

Nine selected SNPs were genotyped, including seven SNPs (rs4613763, rs6880778, rs11742570, rs9292777, rs7725052, rs12186979, and rs10036575) in *PTGER4* and two variants (rs10074991 and rs13361707) in *PRKAA1* (Table 2). The *p*-values of HWE for all selected SNPs in *PTGER4* and *PRKAA1* were > 0.05. The MAFs of rs10074991 and rs13361707 in the *PRKAA1* gene, as well as rs10036575 in the *PTGER4* gene, were lower in GC patients compared to healthy controls (Table 2). Additionally, the SNP rs10074991 (OR = 0.82, 95% CI = 0.69–0.97, *p* = 0.024), rs13361707 (OR = 0.82, 95% CI = 0.69–0.98, *p* = 0.030), and rs10036575 (OR = 0.82, 95% CI = 0.69–0.98, *p* = 0.029) were identified as protective factors for GC susceptibility.

**Table 2:** Details of candidate SNPs and allele model for association between these polymorphisms and GC risk

Genes	SNP-ID	Chr: position	Alleles A/B	MAF		HWE-*p*	OR (95%CI)	*p*	RegulomeDB
				Case	Control				
*PTGER4*	rs4613763	5: 40,392,626	C/T	0.193	0.002	0.999	3.00 (0.60–1.90)	0.158	Other
*PTGER4*	rs6880778	5: 40,398,994	G/A	0.193	0.183	0.140	1.06 (0.85–1.33)	0.599	Other
*PTGER4*	rs11742570	5: 40,410,482	C/T	0.188	0.184	0.182	1.06 (0.85–1.32)	0.625	eQTL/caQTL + TF binding / chromatin accessibility peak
*PTGER4*	rs9292777	5: 40,437,846	T/C	0.268	0.180	0.097	1.05 (0.84–1.32)	0.651	eQTL/caQTL + TF binding / chromatin accessibility peak
*PTGER4*	rs7725052	5: 40,487,168	T/C	0.194	0.261	0.208	1.03 (0.85–1.26)	0.745	eQTL/caQTL + TF binding + any motif + Footprint + chromatin accessibility peak
*PTGER4*	rs12186979	5: 40,524,758	G/A	0.443	0.202	0.494	0.95 (0.76–1.18)	0.631	eQTL/caQTL + TF binding / chromatin accessibility peak
*PTGER4*	rs10036575	5: 40,685,693	C/T	0.443	0.491	0.214	**0.82 (0.69–0.98)**	**0.029**	eQTL/caQTL + TF binding + any motif + Footprint + chromatin accessibility peak
*PRKAA1*	rs10074991	5: 40,790,449	A/G	0.444	0.493	0.287	**0.82 (0.69–0.97)**	**0.024**	eQTL/caQTL + TF binding / chromatin accessibility peak
*PRKAA1*	rs13361707	5: 40,791,782	T/C	0.193	0.492	0.329	**0.82 (0.69–0.98)**	**0.030**	eQTL/caQTL + TF binding / chromatin accessibility peak

GC: gastric cancer; SNP: single nucleotide polymorphism; Chr: chromosome; A: minor alleles; B: major alleles; MAF: minor allele frequency; HWE: Hardy-Weinberg equilibrium; OR: Odds ratio; 95%CI: 95% confidence interval; eQTL: expression quantitative trait locus; caQTL: chromatin accessibility quantitative trait loci; TF: transcription factor

*p* values were calculated from Person’s chi-square test (two-sided)

Bold font and *p* < 0.05 indicates statistical significance

### Overall correlation analysis

Table 3 presents the overall association of the nine selected SNPs with GC susceptibility. The SNP rs10036575 showed a moderate reduction in GC predisposition under the co-dominant (OR = 0.69, 95% CI = 0.49–0.97, *p* = 0.035) and log-additive (OR = 0.83, 95% CI = 0.70–0.98, *p* = 0.034) models. The SNP rs10074991 was found to be a protective SNP against GC occurrence under the co-dominant (OR = 0.68, 95% CI = 0.48–0.96, *p* = 0.028) and log-additive (OR = 0.83, 95% CI = 0.70–0.98, *p* = 0.028) models. Similarly, rs13361707 exhibited decreased odds of GC under the co-dominant (OR = 0.69, 95% CI = 0.49–0.98, *p* = 0.036) and log-additive (OR = 0.83, 95% CI = 0.70–0.99, *p* = 0.034) models.

**Table 3:** Effect of candidate variants on susceptibility to GC

SNP-ID	Model	Genotype	Control (%)	Case (%)	OR (95%CI)	*p*
rs10036575	Co-dominant	TT	138 (27.3)	164 (32.3)	1	**0.035**
		CT	239 (47.2)	238 (46.9)	0.84 (0.63–1.12)	
		CC	129 (25.5)	106 (20.8)	**0.69 (0.49–0.97)**	
	Dominant	TT	138 (27.3)	164 (32.3)	1	0.084
		CC-CT	368 (72.7)	344 (67.7)	0.79 (0.60–1.03)	
	Recessive	CT-TT	377 (82.1)	402 (79.1)	1	0.081
		CC	127 (17.9)	106 (20.9)	0.77 (0.57–1.03)	
	Log-additive	---	---	---	**0.83 (0.70–0.98)**	**0.034**
rs10074991	Co-dominant	GG	136 (26.9)	164 (32.3)	1	**0.028**
		AG	241 (47.6)	239 (47.0)	0.83 (0.62–1.10)	
		AA	129 (25.5)	106 (20.9)	**0.68 (0.48–0.96)**	
	Dominant	GG	136 (26.9)	164 (32.3)	1	0.065
		AA-AG	370 (73.1)	344 (67.7)	0.77 (0.60–1.02)	
	Recessive	AG-GG	377 (74.5)	402 (79.1)	1	0.078
		AA	129 (25.5)	106(20.9)	0.77 (0.58–1.03)	
	Log-additive	---	---	---	**0.83 (0.70–0.98)**	**0.028**
rs13361707	Co-dominant	CC	136 (26.9)	164 (32.2)	1	**0.036**
		TC	242 (47.8)	238 (46.8)	0.82 (0.61–1.09)	
		TT	128 (25.3)	107 (21.0)	**0.69 (0.49–0.98)**	
	Dominant	CC	136 (26.9)	164 (32.2)	1	0.065
		TT-TC	370 (73.1)	345 (67.8)	0.77 (0.59–1.02)	
	Recessive	TC-CC	378 (74.7)	402 (79.0)	1	0.107
		TT	128 (25.3)	107 (21.0)	0.78 (0.58–1.05)	
	Log-additive	---	---	---	**0.83 (0.70–0.99)**	**0.034**

SNP: single nucleotide polymorphism; OR: odds ratio; 95% CI: 95% confidence interval

*p* values were calculated by logistic regression analysis with adjustments for age, gender, smoking, and drinking

Bold font and *p* < 0.05 respects that the data is statistically significant

### Stratification analysis by smoking and adenocarcinoma

Stratification analysis by smoking was performed (Table 4**)**. Among non-smokers, the recessive models showed protective effects of rs10036575 [OR (95% CI) = 0.59 (0.35–0.99), *p* = 0.045], rs10074991 [OR (95% CI) = 0.56 (0.34–0.94), *p* = 0.029], and rs13361707 [OR (95% CI) = 0.58 (0.35–0.97), *p* = 0.037] on the occurrence of GC. Table 5 demonstrates that rs10036575 in the co-dominant (OR = 0.67, *p* = 0.046) and recessive (OR = 0.70, *p* = 0.045) models, as well as rs10074991 in co-dominant (OR = 0.65, *p* = 0.034), recessive (OR = 0.70, *p* = 0.045) and additive (OR = 0.81, *p* = 0.038) models, were significantly associated with a reduced risk of GC adenocarcinoma.

**Table 4:** Association between selected polymorphisms and GC risk according to stratification by smoking

SNP-ID	Model	Genotype	Smoking		Non-Smoking	
			OR (95% CI)	*p*	OR (95% CI)	*p*
rs10036575	Co-dominant	T/T	1	0.507	1	0.837
		C/T	0.86 (0.54–1.35)		1.05 (0.63–1.79)	
		C/C	0.74 (0.43–1.26)		---	
	Dominant	T/T	1	0.347	1	0.556
		C/C-C/T	0.81 (0.53–1.25)		0.87 (0.54–1.40)	
	Recessive	C/T-T/T	1	0.360	1	**0.045**
		C/C	0.81 (0.52–1.27)		**0.59 (0.35–0.99)**	
	Log-additive	---	0.86 (0.66–1.12)	0.259	0.79 (0.59–1.07)	0.131
rs10074991	Co-dominant	G/G	1	0.361	1	0.712
		A/G	0.81 (0.51–1.28)		1.11 (0.65–1.87)	
		A/A	0.72 (0.43–1.24)		---	
	Dominant	G/G	1	0.259	1	0.609
		A/A-A/G	0.78 (0.50–1.20)		0.88 (0.55–1.43)	
	Recessive	A/G-G/G	1	0.424	1	**0.029**
		A/A	0.83 (0.53–1.31)		**0.56 (0.34–0.94)**	
	Log-additive	---	0.85 (0.65–1.11)	0.237	0.79 (0.58–1.06)	0.119
rs13361707	Co-dominant	C/C	1	0.317	1	0.677
		T/C	0.79 (0.42–1.23)		1.12 (0.66–1.90)	
		T/T	0.72 (0.50–1.25)		---	
	Dominant	C/C	1	0.226	1	0.666
		T/T-T/C	0.77 (0.50–1.18)		0.90 (0.56–1.45)	
	Recessive	T/C-C/C	1	0.424	1	**0.037**
		T/T	0.83 (0.53–1.31)		**0.58 (0.35–0.97)**	
	Log-additive	---	0.85 (0.65–1.10)	0.218	0.80 (0.59–1.08)	0.145

SNP: single nucleotide polymorphism; OR: odds ratio; 95% CI: 95% confidence interval: BMI: body mass index

*p* values were calculated by logistic regression analysis with adjustments for adenocarcinoma, smoking, and BMI.

Bold font and *p* < 0.05 respects the data is statistically significant

**Table 5:** Association between selected polymorphisms and the risk of GC according to the adenocarcinoma and other

SNP-ID	Model	Genotype	Adenocarcinoma	
			OR (95% CI)	*p*
rs10036575	Co-dominant	T/T	1	**0.046**
		C/T	0.91 (0.66–1.27)	
		C/C	**0.67(0.45–0.99)**	
	Dominant	T/T	1	0.226
		C/C-C/T	0.83 (0.61–1.13)	
	Recessive	C/T-T/T	1	**0.045**
		C/C	**0.70 (0.50-1.00)**	
	Log-additive	---	0.82 (0.68-1.00)	0.053
rs10074991	Co-dominant	G/G	1	**0.034**
		A/G	0.88 (0.63–1.22)	
		A/A	**0.65 (0.44–0.97)**	
	Dominant	G/G	1	0.153
		A/A-A/G	0.80 (0.59–1.09)	
	Recessive	A/G-G/G	1	**0.045**
		A/A	**0.70 (0.50-1.00)**	
	Log-additive	---	**0.81 (0.67–0.99)**	**0.038**
rs13361707	Co-dominant	C/C	1	0.511
		T/C	0.88 (0.64–1.23)	
		T/T	0.67 (0.45-1.00)	
	Dominant	C/C	1	0.182
		T/T-T/C	0.81 (0.59–1.10)	
	Recessive	T/C-C/C	1	0.068
		T/T	0.73 (0.52–1.02)	
	Log-additive	---	0.82 (0.68-1.00)	0.055

SNP, single nucleotide polymorphism; OR, odds ratio; 95% CI, 95% confidence interval

*p* values were calculated by logistic regression analysis with adjustments for adenocarcinoma, smoking and BMI.

Bold font and *p* < 0.05 respects the data is statistically significant

### MDR analysis

**Fig. 1:**
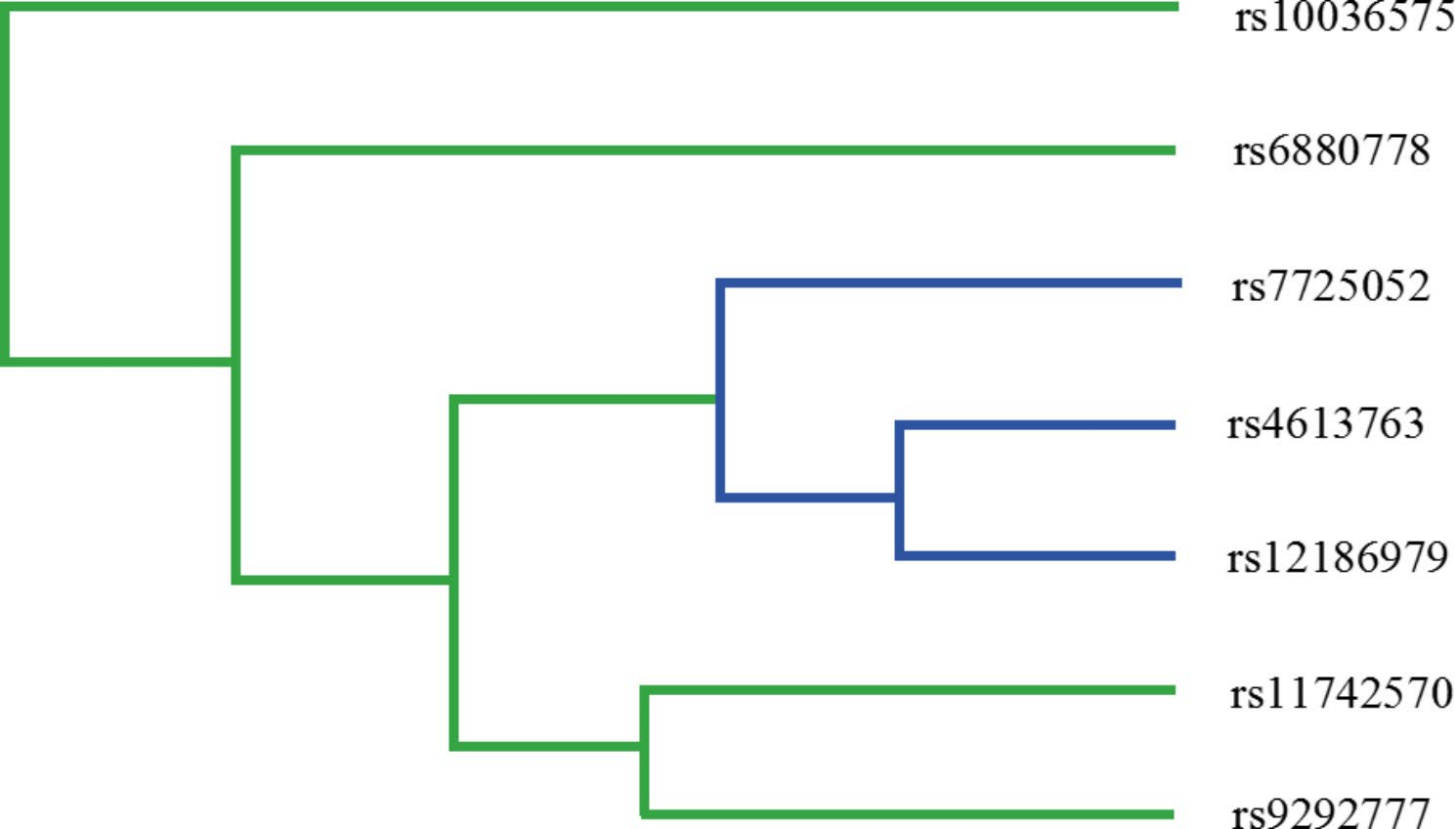
Fruchterman-reingold of MDR analysis of seven candidate SNPs in *PTGER4* The closer to red the stronger the synergy, the closer to the blue the more redundancy

The interaction between seven candidate SNPs in *PTGER4* is illustrated in Fig. 1. Table 6 displays all the experimental results. The best multi-gene locus model for predicting the risk of GC was found to be the seven-variant model: rs4613763, rs6880778, rs11742570, rs9292777, rs7725052, rs12186979, rs10036575 (CVC = 10/10, *p* < 0.001), which is the best multi-gene locus model. The six-SNP model is rs4613763, rs11742570, rs9292777, rs7725052, rs12186979, rs10036575 (CVC = 7/10, *p* < 0.001) and the three-SNP model is rs9292777, rs12186979, rs10036575 (CVC = 6/10, *p* < 0.001) were also better models. Therefore, the impact of the seven candidate SNPs on GC risk may be interdependent.

**Table 6:** SNP–SNP interaction models of candidate SNPs analyzed by MDR method

Model	Training Bal. Acc.	Testing Bal. Acc.	OR(95%CI)	*p* value	CV Consistency
rs10036575	0.527	0.504	1.28 (0.98–1.67)	0.075	10/10
rs9292777, rs10036575	0.540	0.483	1.34 (1.05–1.72)	**0.020**	4/10
rs9292777, rs12186979, rs10036575	0.555	0.480	1.53 (1.19–1.96)	**0.001**	6/10
rs6880778, rs7725052, rs12186979, rs10036575	0.570	0.454	1.69 (1.32–2.18)	**< 0.001**	5/10
rs11742570, rs9292777, rs7725052, rs12186979, rs10036575	0.573	0.456	1.74 (1.35–2.24)	**< 0.001**	5/10
rs4613763, rs11742570, rs9292777, rs7725052, rs12186979, rs10036575	0.574	0.470	1.77 (1.38–2.28)	**< 0.001**	7/10
rs4613763, rs6880778, rs11742570, rs9292777, rs7725052, rs12186979, rs10036575	0.574	0.470	1.78 (1.38–2.28)	**< 0.001**	10/10

MDR: multi-factor dimensionality reduction; Bal. Acc.: balanced accuracy; CVC: cross-validation consistency; OR: odds ratio; 95% CI: 95% confidence interval

Bold values indicate that the value is statistically significant

*p* values were calculated using χ^2^ tests

Bold font and *p* < 0.05: indicates statistical significance

### Association between SNPs and ***PTGER4*** and ***PRKAA1*** expression

**Fig. 2:**
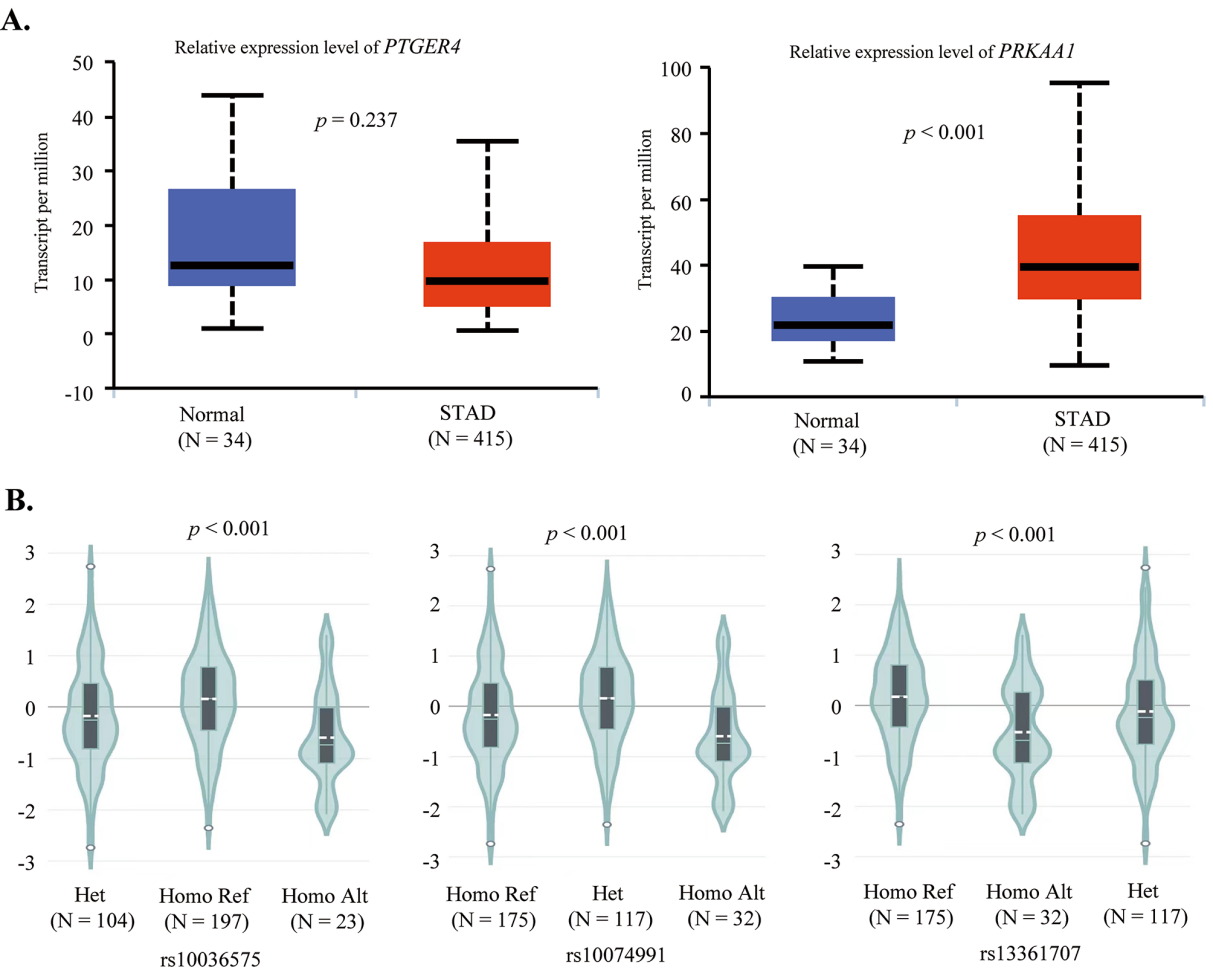
Association between SNPs and *PTGER4* and *PRKAA1* expression **A:** The expression levels of *PTGER4* and *PRKAA1* in STAD and normal tissues; **B:** The different genotypes of SNPs and genes expression levels in stomach Alt: Alternative; Het: Heterozygous; Homo: Homozygous; Ref: Reference; STAD: stomach adenocarcinoma

The prediction results through the Ualcan database showed that the expression of *PRKAA1* in STAD tissue was significantly higher than that in normal tissues (*p* < 0.001) (Fig. 2A). However, no difference was found in the expression level of *PTGER4* between STAD tissue and normal tissue (Fig. 2A). Furthermore, we used the GTEX database to predict the relationship between SNPs and the expression levels of *PTGER4* and *PRKAA1* in the stomach. It was found that there were significant differences in gene expression levels among different genotypes of rs10036575, rs10074991, and rs13361707 (*p* < 0.001, Fig. 2B), indicating that mutations at these loci may affect gene expression.

### ***PTGER4*** and ***PRKAA1*** may affect GC by mediating RhoA

**Fig. 3:**
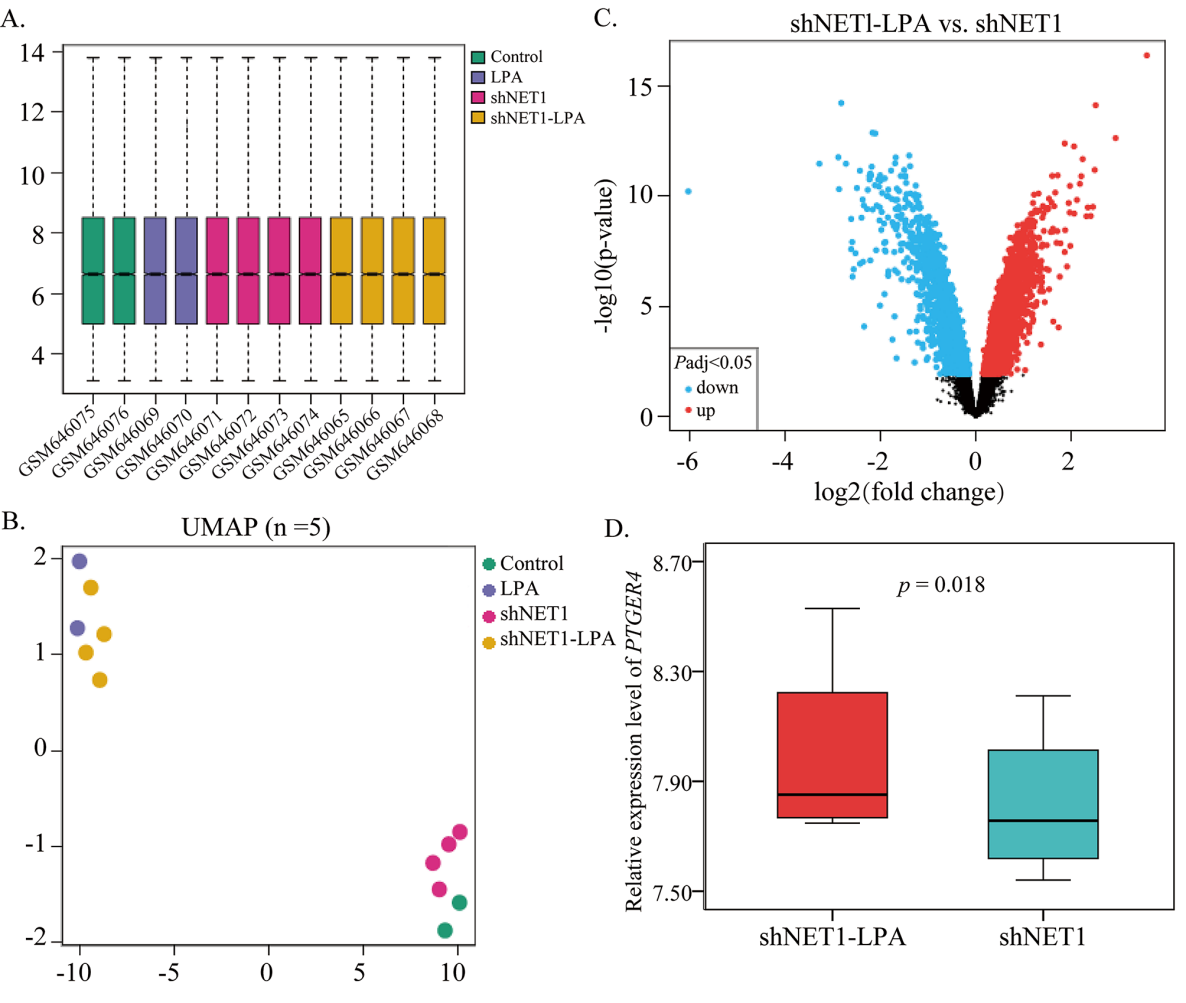
GSE26309 dataset analysis **A:** Grouping and sample size; **B:** Cluster graph; **C:** Volcanic map

**Fig. 4:**
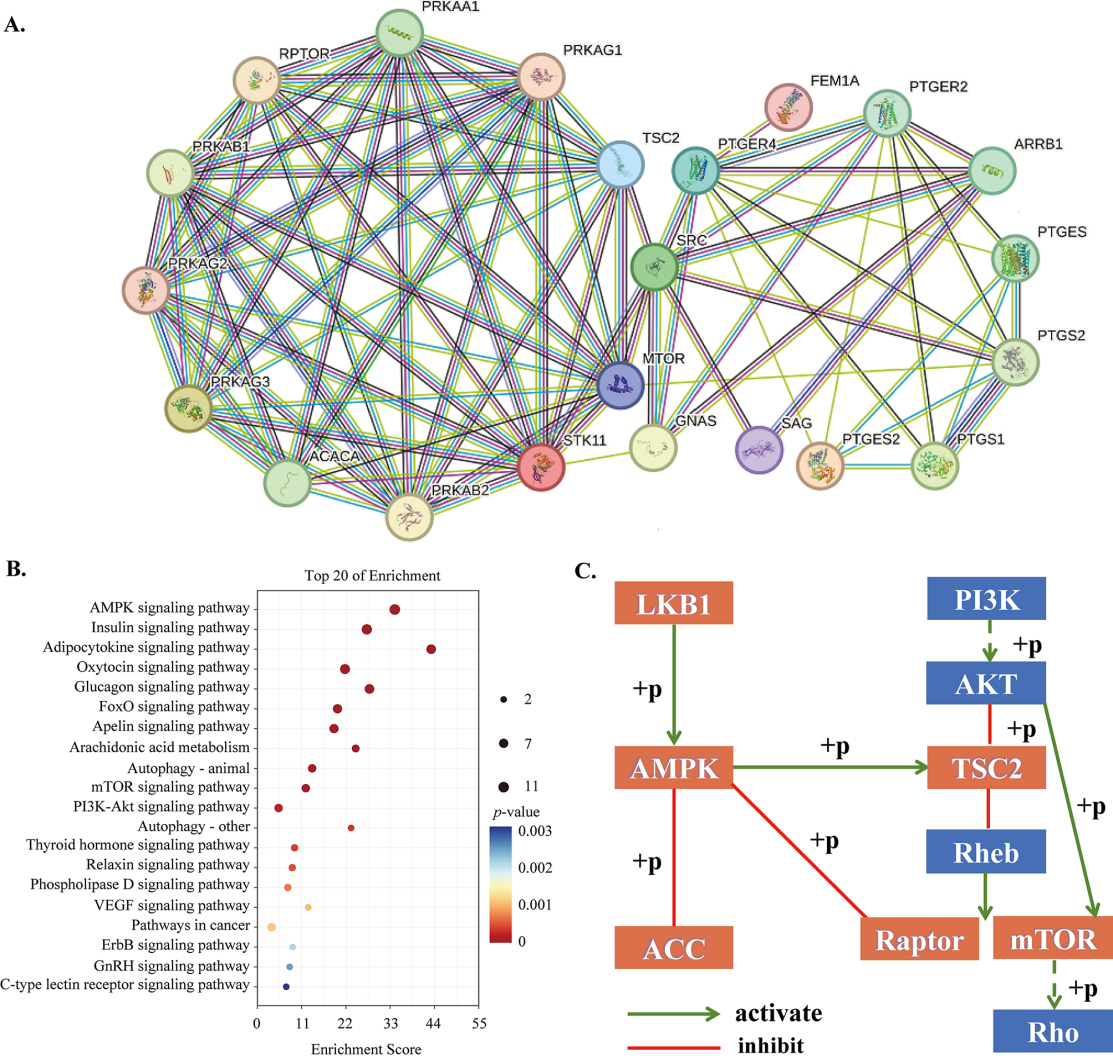
Bioinformatics analysis of *PTGER4* and *PRKAA1* **A:** Protein interaction diagram; **B:** KEGG enrichment results; **C:** Pathway regulation mechanism

Referring to mining of the GEO database, the GSE26309 dataset was divided into AGS gastric cancer cells Control group, RhoA activator group (LPA), RhoA GEF exchange factor (NET1) knockdown group (shNET1), NET1 knockdown and RhoA activator group (shNET1-LPA) (Fig. 3A). Among them, Control group (2 samples) and shNET1 group (4 samples) clustered together, while LPA (2 samples) and shNET1-LPA (4 samples) clustered together (Fig. 3B). The results showed that *PTGER4* expression (*p* = 0.003) was observably increased in LPA group compared with shNET1 group (Fig. 3C). The results of protein interaction map (Fig. 4A) and enrichment analysis (Fig. 4B) indicate that 22 proteins related to PTGER4 and PRKAA1 were involved in the regulation of AMPK, Insulin and Adipocytokine signaling pathway. The pathway mechanism revealed that serine/threonine-protein kinase (STK11, LKB1) activates AMpkase (PRKAA1, PRKABs, and PRKAGs) and thus Acetyl-CoA carboxylase 1 (ACACA), AMpkase can also target tuberin (TSC2) in PI3K/AKT signaling pathway to activate Raptor (RPTOR) and mTOR phosphorylation, thus regulating Rho (https://www.kegg.jp/pathway/map04150, Fig. 4C).

## Discussion

As we all know, the occurrence and development of GC are the result of the combined action of genes and the environment, and the genetic variations are likely to be related to the etiology of GC [27]. The identification of SNPs that can indicate GC is a significant advancement in the prevention and treatment of GC. In-depth exploration of the correlation between these SNPs and GC is of great importance for early detection and prevention of GC. The findings of this study demonstrated that rs10036575 in *PTGER4* and rs10074991 and rs13361707 in *PRKAA1* were significantly associated with a reduced risk of GC. Furthermore, these three SNPs were significantly associated with the expression levels of their corresponding genes (*PTGER4* and *PRKAA1*). Additionally, *PRKAA1* may affect GC through the mediation of RhoA.

*PRKAA1* is the catalytic subunit of the AMPK energy sensor kinase, which regulates cellular energy metabolism through phosphorylation [14]. The AMPK signaling pathways may be involved in the development of GC by participating in cell invasion and metastasis, autophagy and epithelial mesenchymal transformation [28]. Studies have shown that genetic variations of *PRKAA 1*are risk factors for GC [14, 29]. The relationship between *PRKAA1* gene polymorphisms and GC susceptibility has attracted widespread attention from researchers, but the results are not entirely consistent. Numerous reports have indicated that the rs13361707 locus of the *PRKAA1* gene can increase GC risk [18, 29–31], and the rs10074991 locus can increase the risk of gastric cardia and non-cardia GC [32], which contradicts the findings of this study on the population of Hainan province. This discrepancy may be due to differences in the studied population and the limitations of the sample size, and further verification with a larger sample size is required. Stratified analysis suggests a protective effect of *PRKAA1* rs10074991 and rs13361707 on GC in non-smokers, and rs10074991 was also associated with GC adenocarcinoma. Enrichment analysis reveals that *PRKAA1*, as an AMPKase, can participate in the regulation of AMPK and PI3K/AKT/mTOR pathways. In summary, *PRKAA1* gene variation plays a crucial role in GC tumorigenesis.

The protein encoded by *PTGER4* (Prostaglandin E Receptor 4) is a member of the G-protein coupled receptor family and is one of the four receptors identified for prostaglandin E2 (PGE2). Studies have found that *PTGER4* gene locus are associated with various diseases, such as rs4613763 being associated with ulcerative colitis [33, 34]. Among a large number of cancer patients, the mortality rate is significantly higher in smokers compared to non-smokers, such as in lung cancer [35, 36]. These results are consistent with the stratified analysis of *PTGER4* rs10036575 in non-smokers with GC. When BMI ≤ 24 kg/m^2^, *PTGER4* rs10036575 may act as a protective factor for GC, while for BMI > 24 kg/m^2^, it may be associated with susceptibility to GC, suggesting a certain correlation between GC diagnosis and BMI [37]. In this study, *PTGER4* rs10036575 was found to be a protective factor in non-smokers and participants with adenocarcinoma. Moreover, there is a significant correlation between rs10036575 and *PTGER4* gene expression. In conclusion, genetic variations and expression levels of *PTGER4* may influence GC.

However, there are limitations in the present study that should be addressed Firstly, a large proportion of the study sample lacked information on smoking, alcohol consumption, and BMI, which may have influenced the results. Additionally, the lack of information on Helicobacter pylori infection status limits the ability to draw conclusions on the relationship between *PTGER4* and *PRKAA1* polymorphisms and GC risk. Future studies should aim to collect more comprehensive information, including these factors, to obtain a more accurate understanding of the potential relationship between *PTGER4* and *PRKAA1* polymorphisms and GC risk. Finally, this study did not confirm the association between SNPs and gene expression, as well as the specific functional mechanisms. Therefore, further research is required to delve into these matters and gain a more comprehensive understanding.

## Conclusion

Our results demonstrate that the *PTGER4* gene locus rs10036575 and *PRKAA1* gene loci rs10074991 and rs13361707 are associated with GC susceptibility, suggesting that variations in *PTGER4* and *PRKAA1* may affect GC susceptibility. This study also highlights the protective role of *PTGER4* polymorphisms in GC predisposition. These findings provide a new biological perspective for assessing GC risk, exploring its pathogenesis, and developing personalized treatments.

## Data Availability

The datasets used and analyzed during this study are available from the corresponding author on reasonable request.
